# Sleeve Gastrectomy Reduces Glycemia but Does Not Affect Cognitive Impairment in Lean 5xFAD Mice

**DOI:** 10.3389/fnins.2022.937663

**Published:** 2022-08-11

**Authors:** Itia Samuel, Rachel Ben-Haroush Schyr, Yhara Arad, Tamar Attali, Shira Azulai, Michael Bergel, Aviv Halfon, Liron Hefetz, Tamir Hirsch, Hadar Israeli, Neta Lax, Keren Nitzan, Dana Sender, Sahar Sweetat, Eitan Okun, Hanna Rosenmann, Danny Ben-Zvi

**Affiliations:** ^1^Department of Developmental Biology and Cancer Research, The Institute of Medical Research Israel-Canada, The Hebrew University of Jerusalem-Hadassah Medical School, Jerusalem, Israel; ^2^Department of Military Medicine and Tzameret, Faculty of Medicine, The Hebrew University of Jerusalem, Jerusalem, Israel; ^3^The Leslie and Susan Gonda Multidisciplinary Brain Research Center, Bar-Ilan University, Ramat Gan, Israel; ^4^The Paul Feder Laboratory on Alzheimer’s Disease Research, Bar-Ilan University, Ramat Gan, Israel; ^5^The Mina and Everard Goodman Faculty of Life Sciences, Bar-Ilan University, Ramat Gan, Israel; ^6^Department of Neurology, The Agnes Ginges Center for Human Neurogenetics, The Hebrew University of Jerusalem-Hadassah Medical School, Jerusalem, Israel

**Keywords:** 5xFAD, bariatric surgery, Alzheimer’s disease, obesity, cognitive impairment, sleeve gastrectomy, mouse model

## Abstract

Obesity and hyperglycemia are risk factors for cognitive decline and for the development of Alzheimer’s Disease (AD). Bariatric surgery is an effective treatment for obesity that was shown to improve cognitive decline in obese patients. Bariatric surgery was shown to exert weight loss independent effects on metabolic diseases such as type 2 diabetes. We tested whether sleeve gastrectomy (SG), a common bariatric surgery, can affect the cognitive impairment in lean, normoglycemic female 5xFAD mice, a genetic model for AD. 5xFAD mice and wild-type (WT) littermates underwent SG or sham surgery at the age of 5 months and were tested for metabolic, behavioral, and molecular phenotypes 90 days later. SG led to a reduction in blood glucose levels and total plasma cholesterol levels in 5xFAD mice without inducing weight loss. However, the surgery did not affect the outcomes of long-term spatial memory tests in these mice. Analysis of β-Amyloid plaques corroborated the behavioral studies in showing no effect of surgery on the molecular phenotype of 5xFAD mice. In conclusion, SG leads to an improved metabolic profile in lean female 5xFAD mice without inducing weight loss but does not affect the brain pathology or behavioral phenotype. Our results suggest that the positive effects of bariatric surgery on cognitive decline in obese patients are likely attributed to weight loss and improvement in obesity sequelae, and not to weight loss independent effects of surgery.

## Introduction

Maintaining a healthy lifestyle that involves exercise and a balanced diet can delay the development of dementia and cognitive decline (Ahlskog et al., 2011; Ngandu et al., 2015; Spitznagel et al., 2015; Rochette et al., 2016; Stephen et al., 2017). Obesity and hyperglycemia are risk factors for the onset of dementia and Alzheimer’s Disease (AD) (Arvanitakis et al., 2004; Beydoun et al., 2008; Whitmer et al., 2008; Gudala et al., 2013; Alford et al., 2018). Brain imaging and post-mortem biochemical studies demonstrated alteration in glucose metabolism and insulin resistance in the brain of AD patients (Mosconi, 2005; Mistur et al., 2009; Talbot et al., 2012; Yan et al., 2020). Changes in neuronal and glial metabolism, obesity-associated inflammation, an increase in the permeability of the blood-brain barrier, and dysbiosis of the gut microbiome were all implicated in the association between AD and obesity (Kivipelto et al., 2005; De La Monte and Wands, 2008; Pérez-González et al., 2011; De Felice and Ferreira, 2014; Kiliaan et al., 2014; Tucsek et al., 2014; Leigh and Morris, 2020; Sun et al., 2020). The correlation between hyperglycemia and obesity to the risk of developing AD-prompted organizations such as the Alzheimer’s Society in the United Kingdom and the Alzheimer’s Association in the United States to advocate for a healthy lifestyle as means to delay the development of dementia. Yet, there is no specific preventive treatment to reduce the risk for AD, especially in people that do not have a metabolic risk factor (Ahlskog et al., 2011; Brain Health | Alzheimer’s Association, 2022; Staying healthy with dementia | Alzheimer’s Society, 2022).

Bariatric surgery is an effective medical intervention for obesity and its associated comorbidities (Adams et al., 2017; Carlsson et al., 2020). Clinical and animal studies showed that bariatric surgery has weight loss dependent and independent effects on glycemia, cardiovascular disease, and other pathophysiological and physiological processes (Chambers et al., 2011; Sjostrom, 2013; Douros et al., 2019; Li et al., 2019; Aminian et al., 2021; Azulai et al., 2021; Akalestou et al., 2022a; Hefetz et al., 2022). The surgery exerts its effects by restricting the volume of food patients can consume and by changing gut microbial composition, intestinal and hepatic metabolism, gut-brain signaling, and secretion of gastrointestinal and pancreatic hormones (Laferrère, 2011; Saeidi et al., 2013; Arble et al., 2015; Kim et al., 2018; Akalestou et al., 2022b). In particular, bariatric surgery can increase postprandial secretion of the glucagon-like peptide 1 (Glp1), a hormone that augments insulin secretion in response to glucose and induces satiety. Glp1 receptor agonists are used to treating obesity and type 2 diabetes. This treatment is associated with a reduction in the occurrence of cognitive impairment in some observational studies (Gejl et al., 2016; Femminella et al., 2019; Cukierman-Yaffe et al., 2020; Nørgaard et al., 2022). Phase III clinical trials testing the ability of Glp1 receptor agonists to slow the progression of AD are currently underway (ClinicalTrials.gov, 2022).

The weight loss, hormonal changes, and improvement in glycemic control that follow bariatric surgery raise the hypothesis that bariatric surgery can reduce the risk for development of AD in patients with obesity (Stanek and Gunstad, 2013; Keshava et al., 2017; Nota et al., 2020). Most medium-term studies show an improvement in cognitive function years after bariatric surgery (Miller et al., 2013; Alosco et al., 2014a,b; Spitznagel et al., 2015; Rochette et al., 2016; Thiara et al., 2017). However, it is not possible to determine if this improvement is due to weight loss and the associated improvement in glycemia and other sequelae of obesity, or whether the metabolic and endocrine changes triggered by surgery itself (Stefater et al., 2012; Holst et al., 2018; Abidi et al., 2020) contribute to the improvement in cognitive function.

In this study, we sought to test the weight loss independent effect of bariatric surgery on the development of AD-related deficits. To this end, we operated on lean, normoglycemic female 5xFAD mice. The 5xFAD mouse models the development of early onset AD by incorporating human genes with specific mutations in the *APP* and *PSEN1* genes that were shown to cause familial AD (Oakley et al., 2006; Habib et al., 2020; Oblak et al., 2021). Inducing obesity in such models by providing a calorie-dense diet accelerates the development of disease at the molecular and functional levels, and enables research on the interaction between diabetes, obesity, and AD (Lin et al., 2016; Suzzi et al., 2022). We show that while surgery reduces levels of glucose and improves the lipid profile of lean 5xFAD mice without affecting their weight, it does not affect the number of β-Amyloid plaques or the outcomes of long-term memory tests.

## Materials and Methods

### Mouse Strain

5xFAD mice (Oakley et al., 2006) were crossed with C57BL/6J mice and their offspring were genotyped to identify the presence of *APP* and *PSEN1* transgenes. Mice comprising both transgenes were used for the experiments, while their littermates not carrying the transgenes were designated as wild-type (WT) mice. All animal experiments were performed in an AAALAC-approved SPF animal facility under the supervision of the Hebrew University’s Institution Animal Care and Use Committee.

### Sleeve Gastrectomy and Sham Surgery

Five-month-old 5xFAD and WT mice underwent Sleeve Gastrectomy or sham surgery as described previously (Abu-Gazala et al., 2018). In brief, the mice were anesthetized, shaved, and disinfected. Mouse stomach was exposed and a 12-mm clip was placed by using a Ligaclip^®^ Multiple Clip Applier horizontally across the greater curvature of the stomach. The excluded part of the stomach was excised. Sham surgeries included the abdominal incision, stomach mobilization, and closure of the body wall and skin. Mice were fasted the day before surgery and the day of surgery and then returned to normal chow. Mice received analgesics for the 3 days after surgery. Three months after surgery, mice were fasted for 6 h and then were anesthetized before sacrifice.

### Behavioral Assays

T-maze test (Binyamin et al., 2019): The T-maze was used to examine long-term spatial memory. The maze contained two arms of 45 cm in length and 10 cm in width that extended at a right angle from a 57-cm-long alley. The test comprised two trials with an interval of 24 h. On the first day, mice are placed in the start arm of the maze and allowed to explore it for 8 min, while one of the short arms is closed. On the second day, both arms are open and the animal is allowed to explore all maze parts for 3 min. The number of entries to the unfamiliar arm and the time spent there were recorded.

The radial arm water maze (RAWM) (Alamed et al., 2006): The RAWM was used to examine spatial learning and long-term memory. The maze contained six swim paths (arms) extending out of an open central area, with an escape platform located at the end of one arm—the goal arm. The goal arm location remained constant for each mouse. On day one, mice are trained for 15 trials spaced over 3 h, with trials alternating between visible and hidden platforms. On day two, mice are trained for 15 trials with the hidden platform. Entry into an incorrect arm is scored as an error. The results are presented in blocks of three consecutive trials.

Open field habituation test (Binyamin et al., 2019): The test was used to examine long-term spatial memory. The test measures the decrease in the exploratory activity of the animal in a test session carried out 24 h after the first exploration session. On the training day, animals were exposed to a novel environment: placed in a 40 cm × 50 cm × 60 cm open field box for a 5-min period. Twenty-four hours later, animals were re-exposed to the same environment. Locomotion on the training and testing days was recorded using the Ethovision10 system, providing computerized, blinded, and unbiased measurement.

### Histology

Brains were placed in 4% PFA/PBS overnight. The brains were then washed three times for 5 min with 4°C PBS and then placed in 30% sucrose/PBS overnight after which the tissue sunk in the sucrose/PBS solution. The brains were frozen in OCT (Tissue Tek #4583) and stored at –80°C. The brains were cryo-sectioned sagittally at a thickness of 10 μm and stored at –80°C.

Histochemistry: Slides were stained with 1% Thioflavin-T/DDW (T3516 Sigma) in the dark for 5 min and then washed with 70% ethanol for 5 min, followed by three 5-min washes with DDW.

Immunohistochemistry: Slides were blocked with Triton-X 0.2%/Cas block (ThermoFisher 008120) for 1 h and then incubated with the primary antibody for 40 h at 4°C. The primary antibody solution contained Triton-X 0.2%/Cas block. The following antibodies were used: mouse anti-humanβ-Amyloid (clone 6E10, BioLegend 803004) 1:375; mouse anti-mouse GFAP (Cell Signaling 3670) 1:200; and rabbit anti-mouse Iba1 (Wako/FUJIFILM 019-19741) 1:750. Slides were then washed three times with PBST for 5 min and incubated with a secondary antibody in a 1%BSA/PBS solution for 2 h at room temperature (Jackson ImmunoResearch 711-165-152, 715-605-151) 1:500. Before closing the slides with a mounting medium (DiaSorin, 2505-5), we incubated slides with DAPI (Sigma-Aldrich, Cat. No. D9564 20 μg/ml final concentration diluted in DDW).

Image quantification: Fluorescence intensity of Iba1 and GFAP were quantified using ImageJ by applying a threshold for background fluorescence. The number of positive pixels/unit area was normalized to 1 in the 5xFAD sham animals. The number of immunofluorescent β-amyloid spots and Thioflavin T positive plaques were counted per unit area.

### Quantification of Hippocampal β-Amyloid_42_ Levels

Hippocampal fibrillar β-amyloid (Aβ) was extracted and measured using a modification of a sandwich-ELISA protocol for Aβ peptides (Illouz et al., 2017). In brief, tissues were mechanically homogenized in RIPA buffer [150 mM NaCl, 5 mM EDTA, pH 8.0, 50 mM Tris, pH 8.0, 1% Triton, 0.5% Na-Deoxycholate, 0.1% SDS, 150 mg/ml (tissue/buffer)], including protease inhibitor cocktail (1:100, Sigma P2714), then incubated on ice for 30 min followed by centrifugation for 120 min at 17,000 × g at 4°C. The supernatant, containing soluble Aβ was removed and stored at –20°C. The remaining pellet, containing insoluble Aβ was resuspended in 70% formic acid and incubated on ice for 30 min, followed by centrifugation for 120 min at 17,000 × g at 4°C. The formic acid supernatant was separated and neutralized using 1M Tris (pH = 11, 20-time the volume of the formic acid) and stored at –20°C. Total protein concentration was determined using the BCA method (Thermo Fisher Scientific, 23225). Sandwich-ELISA was performed in 96-well polystyrene microplates (Greinerbio-one, 655061) covered with 50 μl of mouse anti-N-terminus Aβ16 (clone 6E10, Biolegend, 803002) at a concentration of 2.5 μg/ml (1:400) in carbonate–bicarbonate buffer (pH = 9.6) and incubated overnight at 4°C. Plates were washed three times in PBS-T solution (0.05% Tween in PBS) and blocked with 1% BSA solution in PBS-T. A total of 50 μl of tissue formic acid fraction homogenate, diluted 1:5 in 1% BSA solution in PBS-T, was applied to each well, and incubated for 120 min at room temperature. Plates were then washed five times in PBS-T, and the following detection antibodies were added: Rabbit Anti-C terminus-Aβ33–42 (clone 1-11-3, Biolegend, 812101) diluted at 1:2,000 (0.25 μg/ml), and incubated for 120 min at room temperature. Next, plates were washed five times in PBS-T and a secondary goat anti-Rabbit IgG HRP conjugated antibody was added (Peroxidase AffiniPure Goat AntiRabbit, Jackson immunoresearch 111-035-003) at a dilution of 1:10,000. Plates were washed five times with PBS-T and 50 μl of 3, 3′, 5, 5′-tetramethylbenzidine substrate (Affymetrix eBioscience, #00-4201-56) was added. The color reaction was allowed to develop for 3 min and was quenched by adding 50 μl of 2M H2SO4. Optical density was measured at 450 nm using a spectrophotometer. A standard curve was generated using known concentrations of recombinant Aβ_42_.

### Metabolic Profile

Blood glucose was measured with a Roche Accu-Check glucometer by tail bleeding. Non-fasting blood glucose was measured in the first 70 days after surgery, at 7–8 a.m. when animals had *ad libitum* access to food and water throughout the night.

Animals were fasted for 6 h at the end of the experiment, 90 days after surgery. Approximately 800 μL of blood were extracted *via* terminal bleeding using heparin-coated syringes and 25G needles and transferred to lithium heparin-coated tubes (Greiner, MiniCollect, 450535). Blood was then centrifuged at 6,000 RCF for 1.5 min. Plasma was collected and flash-frozen in liquid nitrogen until transferred to final storage at –80°C. Plasma samples were analyzed using COBAS C111 (Roche Diagnostics) for levels of high-density lipoproteins (Roche Diagnostics HDL, 07528604190), low-density lipoproteins (Roche Diagnostics LDL 07005806190), total cholesterol (Roche Diagnostics, 04718917190), and Triglycerides (Roche Diagnostics, 04657594190). Insulin was measured using Ultra-Sensitive Mouse Insulin ELISA (Crystal Chem, 90080).

### Data Analysis

#### Statistics

Three-way repeated measures ANOVA, 2-way ANOVA, or Student’s *t*-test were used to identify an effect of surgery or genotype on metabolic, behavioral, or molecular assays as detailed in the text. Tukey HSD *post-hoc* test was performed if a difference was found. The Holm-Sidak correction was applied to correct for multiple hypotheses testing. Outliers were defined by a difference of more than two standard deviations and were detected only in some behavioral assays. Principal component analysis (PCA) was performed in MATLAB. Groups were considered separable by PCA if a support vector machine could separate the groups with over 80% accuracy.

#### Box Plots

The box presents the range between the first and third quartile in the data. The horizontal line is the median and × denotes the mean. The whiskers correspond to the minimal and maximal data point within the range of the first quartile minus 1.5 times the difference between the third and first quartiles (interquartile range, IQR) and third quartile plus 1.5 times the IQR. Data points outside this range are considered as dots in the plot but are included in the statistical analysis.

## Results

### Sleeve Gastrectomy Affects Metabolic Parameters in Lean 5xFAD Mice and Their Wild-Type Littermates

**FIGURE 1:**
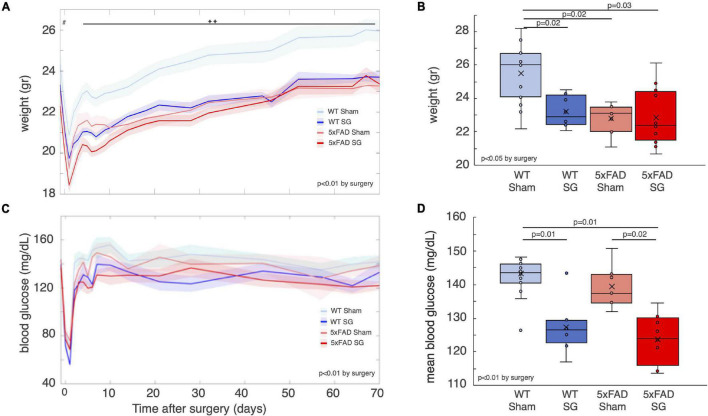
Weight and glycemia of wild-type (WT) or heterozygous 5xFAD mice (5xFAD) following sham surgery or sleeve gastrectomy (SG). **(A)** Average weight of WT and 5xFAD littermates that underwent SG or sham surgery from the day before surgery to 10 weeks after surgery. ^#^*p* = 0.03, 2-way ANOVA by genotype. ^◆◆^*p* < 0.01 between WT sham and three other groups, Tukey *post hoc* analysis following 3-way continuous measures ANOVA. Time was found as a factor in the 3-way ANOVA as well, *p* < 0.01. **(B)** Weight of mice at the end of the experiment, before sacrifice, and 90 days after surgery. **(C)** Blood glucose levels of the four experimental groups. Glucose was measured approximately once a week during the morning while mice were fed *ad libitum*. 3-way repeated measures ANOVA showed a difference by surgery type, as indicated in the figure. **(D)** Mean blood glucose levels from day 10 until day 70 after surgery. Mean levels of glucose in that time period were calculated for each mouse. Shaded areas in **(A,C)** denote the standard error. The *p*-values in **(B,D)** were calculated by Tukey HSD *post hoc* test following 2-way ANOVA. *p* > 0.05 if not indicated. *n* = 13,7,8,12.

Five-month-old lean female 5xFAD mice and their wild-type littermates were randomized into SG or sham surgery. The average pre-surgical weight of 5xFAD mice was 1 g less than WT mice, consistent with previous reports (Gendron et al., 2021; Figure 1A). Non-fasting blood glucose and weight were measured weekly for 70 days after surgery, before behavioral tests. WT sham-operated mice were approximately 10% heavier than SG-operated WT mice and SG and sham-operated 5xFAD mice throughout the experiment (Figure 1A). At the end of the experiment, 90 days after surgery, both SG and sham-operated 5xFAD mice weighed less than WT sham-operated mice and had the same weight as WT SG-operated mice (Figure 1B). SG-operated WT and 5xFAD mice had lower non-fasting blood glucose than sham-operated WT and 5xFAD mice. Genotype did not affect glycemia before or after surgery (Figures 1C,D).

**FIGURE 2:**
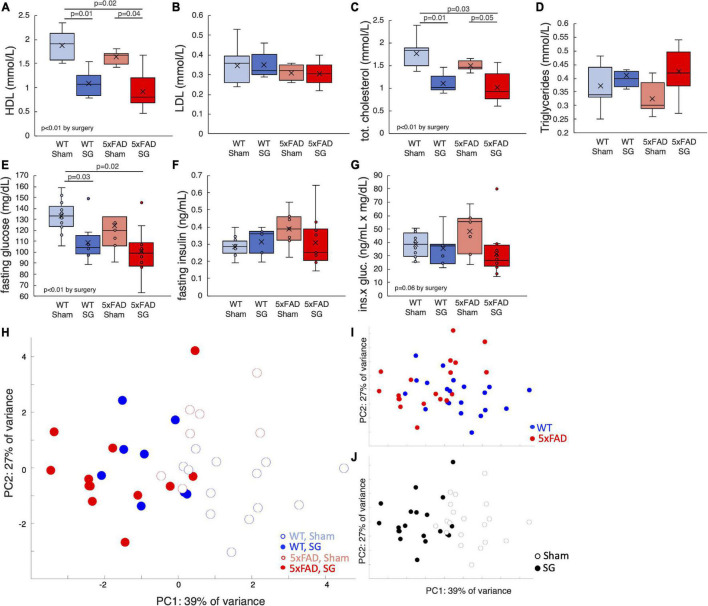
Metabolic parameters of wild-type (WT) or 5xFAD mice following sham surgery or sleeve gastrectomy (SG). **(A–D)** Fasting concentrations of high-density lipoproteins (HDL) **(A)**, low-density lipoproteins (LDL) **(B)**, total cholesterol **(C)**, and triglycerides **(D)** in the plasma. **(E–G)** Fasting concentrations of blood glucose **(F)**, plasma insulin **(G)**, and the product of glucose and insulin **(H)**, which is a proxy for insulin sensitivity. **(H)** Principal component analysis (PCA) of metabolic parameters presented above and weight at the end of the experiment. Each point represents a single mouse. **(I,J)** Same plot as in H, color-coded according to surgery type **(I)** and genotype **(J)**. The *P*-values in calculated by Tukey HSD *post-hoc* test. *p* > 0.05 if not indicated. Two-way ANOVA outcomes as indicated in the figure. *n* = 12–13,6–7,7–8,11–12.

Mice fasted for 6 h at the end of the experiment, and the composition of their plasma was analyzed. SG-operated of both genotypes had lower levels of high-density lipoproteins (HDL) and total cholesterol. There were no differences in the fasting levels of low-density lipoproteins (LDL) or triglycerides between experimental groups (Figures 2A–D). Fasting blood glucose levels of SG-operated mice were lower, yet fasting plasma insulin levels were not affected by surgery (Figures 2E,F). The product of fasting insulin and glucose levels, which reflects insulin resistance, was marginally higher in sham-operated WT and 5xFAD mice (Figure 2G). With the exception of weight, which was lower in 5xFAD mice pre-surgery, SG had a similar effect on WT and 5xFAD operated mice. A principal component analysis (PCA) of the metabolic parameters of the mice at the end of the experiment showed that surgery type separated the mice well, while mice of both genotypes were similarly distributed (Figures 2H–J). We conclude that SG exerts a metabolic effect on lean female WT and 5xFAD mice 3 months after surgery.

### Sleeve Gastrectomy Has no Effect on Spatial Memory in Behavioral Tests

**FIGURE 3:**
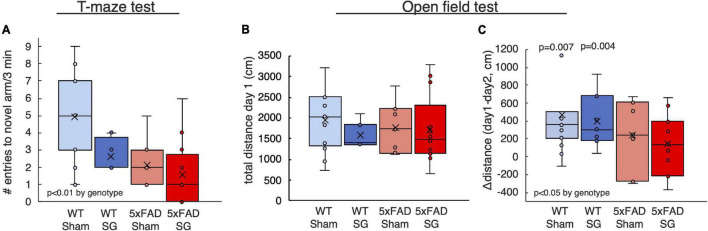
Outcomes of behavioral tests performed on wild-type (WT) or 5xFAD mice following sham surgery or sleeve gastrectomy (SG). **(A)** Number of entries into the novel arm in the T-maze test. **(B,C)** Total distance traveled on the first day of test **(B)**, and difference in distance between the first and second days of the open-field test **(C)**. The *p*-values in **(C)** calculated by single-sample *t*-test on the difference between the first and second day. *p* > 0.05 if not indicated. 2-Way ANOVA outcomes as indicated in the figure. *n* = 11–13,5–7,7–8,11–12.

Mice were subjected to a series of behavioral assays testing their long-term memory at the age of 7 months. The T-maze test is a 2-day test in which the mice are allowed to enter only one arm of a “T” shaped maze on the first day. On the following day, the mice can choose which arm to explore. Healthy mice tend to explore novel arm indicating that they remember entering the arm available on the previous day. 5xFAD mice displayed a characteristic reduction of entries into the novel arm suggesting a reduction in long-term spatial memory. Surgery type did not affect the outcomes of this test (Figure 3A).

The open-field test similarly measures the change in the behavior of mice when introduced into an environment they explored the previous day. Healthy mice will not explore thoroughly a familiar environment. Accordingly, WT but not 5xFAD mice reduced their walking distance on the second day of the test, indicating a reduction in long-term spatial memory in 5xFAD mice. Surgery type did not affect other measures of the open-field test (Figures 3B,C).

**FIGURE 4:**
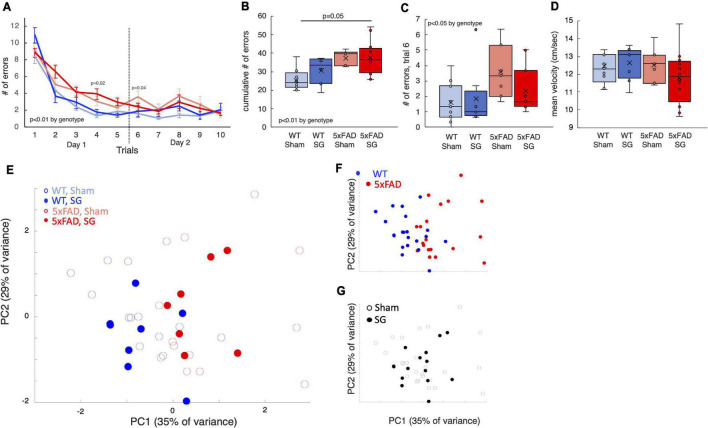
Outcomes of radial arm water maze tests performed on wild-type (WT) or 5xFAD mice following sham surgery or sleeve gastrectomy. **(A)** Duration of trial as a function of trial number. The dashed black line separates the first and second day of the tests. 3-way ANOVA showed a difference in genotype, *p* = 0.003. A significant difference according to time was observed as well, *p* < 0.001. Individual *p*-values on days 4 and 6 mark a difference according to genotype by 2-way ANOVA on a specific day. No difference was found on other days. **(B)** Box plot for the cumulative time of all trials. **(C)** Box plot of trial durations during the first trial of the second day of the test. **(D)** Box plot of the mean velocity during all trials. **(E)** Principal component analysis (PCA) of outcomes of behavioral tests in which there was a statistically significant difference. Each point represents a single mouse. **(F,G)** Same plot as above, color-coded according to surgery type **(F)** and genotype **(G)**. The *p*-values in calculated by Tukey HSD *post-hoc* test. *p* > 0.05 if not indicated. *n* = 12,6,8,12.

In the radial arm water maze (RAWM), mice swim and search for a platform they can stand on. Healthy mice learn rapidly to identify the location of this platform. This test is conducted over 2 days and measures spatial learning and long-term memory. 5xFAD mice made more errors than WT mice in the RAWM (Figures 4A,B), in particular, in the first trial of the second day, indicating a reduction in long-term memory (Figure 4C). Surgery type did not affect the results.

SG did not affect walking distance in the open-field test (Figure 3B) or swimming velocity in the RAWM (Figure 4D), affirming that SG-operated mice had no impairment in movement in these tests. PCA showed that genotype separates well between the groups of mice, while surgery type had no effect (Figures 4E–G). Taken together, 5xFAD mice had worse results in behavioral tests than WT littermates, and surgery type did not affect the results in the assays we performed.

### Sleeve Gastrectomy Does Not Activate Microglia or Astrocytes in the Hippocampus

**FIGURE 5:**
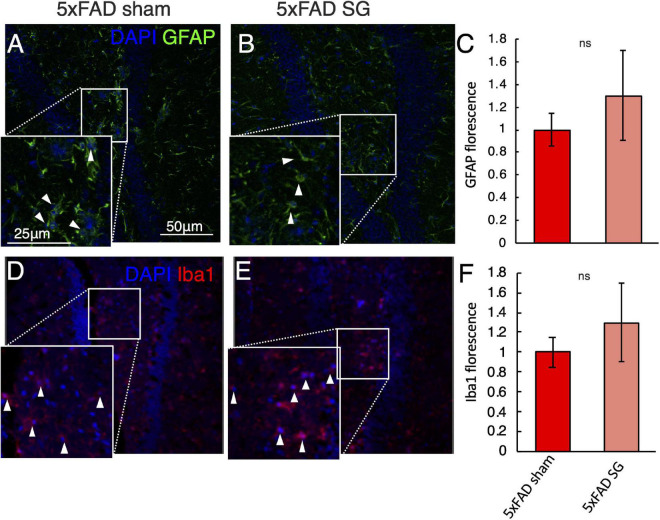
Immunohistochemical analysis of the hippocampus in wild-type (WT) or 5xFAD mice following sham surgery or sleeve gastrectomy (SG). **(A–C)** Representative immunohistochemical staining of GFAP (green) marking astrocytes, and DAPI (blue) marking DNA in the hippocampus of 5xFAD sham **(A)**, 5xFAD SG **(B)**. White arrowheads point to GFAP positive cells in insets. Quantification of GFAP fluorescence is shown in **(C)**. **(D–F)** Representative immunohistochemical staining of Iba1 (red) marking microglia, and DAPI (blue) marking DNA in the hippocampus of 5xFAD sham **(D)**, 5xFAD SG **(E)**. White arrowheads point to Iba1 positive cells in insets. Quantification of Iba1 fluorescence is shown in **(F)**. All images and insets in the same magnification as in **(A)**. ns: not significant by Student’s *t*-test. *n* = 5–7. Error bars in C and F indicate standard error.

The behavioral tests showed a reduction in long-term spatial memory in 5xFAD mice. We, therefore, tested for histological changes in the dentate gyrus of the hippocampus of 5xFAD. GFAP immunostaining showed astrocyte activation in 5xFAD mice with no effect on surgery type (Figures 5A–C). Similarly, surgery type did not affect Iba1 staining in 5xFAD mice (Figures 5D–F).

### Sleeve Gastrectomy Has no Effect on the Number of β-amyloid Plaques in 5xFAD Mice

**FIGURE 6:**
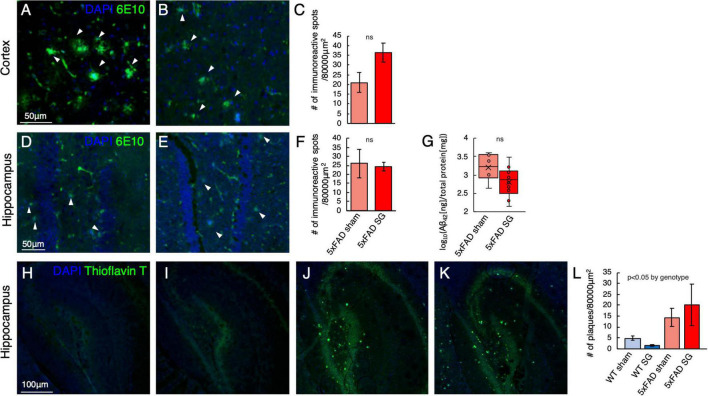
Histological analysis for the presence of β-amyloid plaques in the cortex and hippocampus in wild-type (WT) or 5xFAD mice following sham surgery or sleeve gastrectomy (SG). **(A–C)** Representative immunohistochemical staining of 6E10 antibody, marking human β-amyloid (green), and DAPI (blue) marking DNA in the cortex of sham-operated **(A)** and SG-operated **(B)** 5xFAD mice. Quantification of the number of immunoreactive spots, marking β-amyloid plaques, is shown in **(C)**. **(A,B)** Are in the same magnification as **(A)**. *n* = 5,7. **(D–F)** Representative immunohistochemical staining of 6E10 antibody, marking human β-amyloid (green), and DAPI (blue) marking DNA in the hippocampus of sham-operated **(D)** and SG-operated **(E)** 5xFAD mice. Quantification of the number of immunoreactive spots, marking amyloid-beta plaques, is shown in **(F)**. **(D,E)** Are in the same magnification as **(A)**. *n* = 5,7. **(G)** The log_10_ of the ratio between the concentration of the insoluble fraction of human β-Amyloid_42_, measured in ng, to total protein in the sample, measured in mg. *n* = 6,10. **(H–L)** Representative Thioflavin T-staining (green) marking β-amyloid plaques and DAPI staining for DNA in the hippocampus of WT sham **(H)**, WT SG **(I)**, 5xFAD sham **(J)**, and 5xFAD SG **(K)**. Quantification of the number of plaques in **(L)**. **(H–K)** Are in the same magnification. *n* = 5–7 in all groups. ns: not significant by Student’s *t*-test **(C,F,G)**. 2-way ANOVA **(L)** outcomes are indicated in the figure. Error bars in **(C,F,L)** indicate standard error.

Immunohistochemical analysis using 6E10 antibody for human β-amyloid, expressed in 5xFAD but not in WT mice, showed accumulation of β-amyloid in both cortex (Figures 6A–C) and hippocampus (Figures 6D–F) of 5xFAD mice. Surgery type did not affect both the cortex and hippocampus. There was also no difference in the levels of the insoluble fraction of β-amyloid, which is the fraction that generates the amyloid plaques, between the hypothalami of sham and SG-operated 5xFAD mice (Figure 6G). Histochemical staining for amyloid plaques using Thioflavin T identified a high number of plaques in the hippocampus of 5xFAD mice in both sham and SG-operated 5xFAD mice with little or no staining in WT mice (Figures 6H–L). Surgery type did not affect the number of plaques in either genotype.

## Discussion

Bariatric surgery affects several obesity-associated diseases in most cases (Adams et al., 2017; Carlsson et al., 2020). Clinical and animal studies demonstrated that these effects are mediated by weight loss dependent and independent mechanisms (Chambers et al., 2011; Sjostrom, 2013; Douros et al., 2019; Aminian et al., 2021; Azulai et al., 2021). Some of the positive and negative effects of bariatric surgery were observed also in lean rodents, suggesting that the metabolic and endocrine state induced by surgery can have beneficial or detrimental physiolgoical effects (Li et al., 2019; Ben-Haroush Schyr et al., 2021; Akalestou et al., 2022a; Hefetz et al., 2022). Obesity is associated with cognitive decline and is a risk factor for AD (Ahlskog et al., 2011; Ngandu et al., 2015; Spitznagel et al., 2015; Rochette et al., 2016; Stephen et al., 2017), and bariatric surgery was shown in most studies to improve cognitive function in patients with obesity (Miller et al., 2013; Alosco et al., 2014a,b; Spitznagel et al., 2015; Rochette et al., 2016; Thiara et al., 2017; Prehn et al., 2020; Morledge and Pories, 2021). This result can have two, non-mutually exclusive explanations: First, weight loss and improvement in metabolic parameters that are affected directly by obesity such as glycemia can reverse or delay cognitive decline. Second, the endocrine and metabolic state instigated by bariatric surgery. For example, hypersecretion of gastrointestinal and pancreatic hormones and a different level of specific amino acids, bile acids, and lipids in the serum (Stefater et al., 2012; Holst et al., 2018; Abidi et al., 2020) can impede the progression of the disease.

We sought to test the second hypothesis, namely that bariatric surgery has weight-loss independent effects on cognitive function, by measuring the metabolic, behavioral, and molecular phenotypes of SG on lean 5xFAD mice, a model for AD. This approach allows for isolating surgery-specific effects. As we discuss below, our findings suggest that the main effect of bariatric surgery on cognitive function may be attributed to weight loss, and improvement in the sequelae of obesity—and not to the unique endocrine/metabolic outcomes of surgery.

SG led to weight loss in WT mice, but not in 5xFAD mice. 5xFAD mice were leaner before surgery (Gendron et al., 2021), and both sham and SG 5xFAD mice were leaner than WT sham-operated mice throughout the experiment. SG resulted in a reduction in mean non-fasting levels of glucose in both WT and 5xFAD mice. Fasting blood glucose levels of SG-operated mice were lower. SG also led to a reduction in total cholesterol levels. SG had overall similar results in WT and 5xFAD mice, providing evidence for a weight loss independent effect of surgery on systemic metabolism in lean female mice. The metabolic effect of surgery is demonstrated using a PCA plot of metabolic parameters (Figures 2I–K), which separates well the mice according to surgery type across the first component, while genotype does not separate the data.

Our experimental design enabled us to test how a reduction in glucose and cholesterol levels within the normal range can affect the phenotype of 5xFAD mice, outside the context of obesity and glucose intolerance. This design is suitable for the 5xFAD model, in which brain glucose uptake is affected in lean mice, corroborating findings in patients (Oblak et al., 2021). It also decouples between the positive effects of bariatric surgery on cognitive function associated with improvement in obesity in its sequelae and the metabolic and endocrine effects of surgery which may or may not affect cognitive function.

We observed a behavioral phenotype in the T-maze, open field, and radial arm water maze tests in 5xFAD mice at the age of 8 months. The same phenotype was observed in SG and sham-operated 5xFAD mice, implying that SG has no effect on spatial memory in this model. Importantly, SG did not affect WT mice, validating that the surgery itself did not change the ability of mice to move or modified behavior related to the assays we used.

Histological assays quantifying β-amyloid pathology plaques in the hippocampus and cortex of mice corroborated the results of the behavioral assays. We could not detect a difference between SG and sham-operated 5xFAD mice using immunohistochemical staining for human β-amyloid, using an ELISA for the insoluble fraction of human β-Amyloid, or by Thioflavin T histochemical staining for mature plaques in the mouse brain. Similarly, we did not detect a difference in immunostaining for Iba1, marking microglia and GFAP, and marking astrocytes between SG and sham-operated 5xFAD mice.

Maintaining a healthy lifestyle that results in normoglycemia and normal weight is recommended to delay and perhaps prevent the development of age-related cognitive decline. There is a major effort to develop and repurpose metabolic drugs to treat cognitive decline and AD (Clarke et al., 2018). It is, therefore, reasonable to speculate that lower glucose and cholesterol levels will reduce the risk for dementia and AD. The results we present show that SG performed in lean female mice do not affect the behavioral or molecular phenotype of 5xFAD mice, even though lower glycemia and cholesterol were achieved. An interpretation of these results is that the correlation between glycemia and the risk of AD may not extend within the normal ranges of glucose and cholesterol. Notably, the benefits of exercise, a balanced diet, and a healthy lifestyle extend beyond measures such as weight and glycemia and can reduce the risk of disease without affecting glucose levels, lipid profile, or weight.

We studied the effects of SG on AD in a specific genetic model, in females, and in a single age and time after surgery. A different model, time, or assay could have different results. We used lean female mice to minimize the effect of surgically induced weight loss; male 5xFAD mice weigh more than females and were likely to lose weight following surgery, making interpretation of weight loss independent effects difficult. In our hands, a behavioral phenotype is robustly detected at the age of 7 months in the 5xFAD model. We chose to operate 2–3 months before the tests to minimize short-term outcomes of surgery related to recovery, which can affect behavior even in WT mice, but not too long before the behavioral tests, as the effects of surgery may wane.

Accumulation of β-amyloid plaques and metabolic changes in the hippocampus of 5xFAD mice were reported already at the age of 5 months (Binyamin et al., 2019; Oblak et al., 2021), when we operated on mice. It is arguable that at this stage, it is too late to change the course of the disease in this model. Although we could detect a reduction in glucose and cholesterol levels in SG-operated mice, the effect may be too small to have measurable consequences on cognitive decline. Furthermore, behavioral and molecular assays have high variability within each group. Altogether, a greater number of animals could have provided greater power to identify an effect of SG on 5xFAD mice. Nonetheless, in most assays, the effect of the size of surgery was very small. Accordingly, PCA of the behavioral assays separated the mice by genotype and not by surgery type.

It would be important to test if SG can improve cognitive function in an obese model for AD, such as 5xFAD mice fed on a high-fat diet. Failure of SG to improve cognitive function in that model will imply that the genetic disposition in these models cannot overcome by surgically induced weight loss, and therefore this model is inadequate to address the effects of SG on cognitive decline. Improvement in cognitive function will support the hypothesis that cognitive decline can be delayed by weight loss and improvement in metabolic parameters independent of weight loss.

We were not successful in measuring the post-prandial levels of Glp1 in the plasma of mice following SG or sham surgery in this experiment for technical reasons. However, we and others have shown that sleeve gastrectomy is associated with an elevation in the levels of Glp1 in obese patients and in obese and lean rodents (Chambers et al., 2011, Wilson-Pérez et al., 2013; Akalestou et al., 2022b; Hefetz et al., 2022). Patients treated with Glp1 receptor agonists for hyperglycemia had a lower occurrence of cognitive impairment (Gejl et al., 2016; Femminella et al., 2019; Cukierman-Yaffe et al., 2020; Nørgaard et al., 2022). Glp1 has neuroprotective effects which may contribute to a positive role of this hormone in AD (Erbil et al., 2019). Our findings here suggest that within this experimental setting, the effect of Glp1 may be small.

An important limitation of this study is that it employs a genetic model of familial AD. To date, there is no reliable rodent model for sporadic AD which is the more common form of AD. We and others have shown that SG is not effective in maintaining weight loss in early-onset genetic obesity modeled by db/db mice or Melanocortin 4 Receptor null rats, yet it is effective in obesity models induced through a calorie-rich diet (Hatoum et al., 2012; Abu-Gazala et al., 2018; Ben-Haroush Schyr et al., 2021). Our data, therefore, do not reject the hypothesis that SG can have a weight loss independent effect in sporadic AD in patients, and that this effect cannot overcome the strong genetic basis of disease in 5xFAD mice.

## Conclusion

In conclusion, we show that SG has no effect on the AD-related behavioral or molecular phenotype in lean female 5xFAD mice. SG was effective in reducing glycemia and levels of cholesterol within the normal range in WT and 5xFAD mice, but the surgery did not affect behavioral or histological assays in 5xFAD mice. Surgery type did not affect the locomotion of WT mice or have any other effect on their behavior. Our results suggest that a positive effect of bariatric surgery on cognitive decline in patients is mediated primarily by weight loss and consequential improvement in metabolic parameters, and not *via* surgery-specific changes in endocrine signaling or metabolic regulation. Moreover, a reduction in glucose and cholesterol levels within the normal range may not have a beneficial effect on the pathogenesis of AD.

## Data Availability Statement

The raw data supporting the conclusions of this article will be made available by the authors, without undue reservation.

## Ethics Statement

The animal study was reviewed and approved by IACUC committee of Faculty of Medicine in The Hebrew University of Jerusalem.

## Author Contributions

IS, KN, RB-HS, HR, and DB-Z conceived the study. IS, RB-HS, and DB-Z operated on mice. IS, RB-HS, YA, SA, MB, LH, and HI contributed to the post-operative treatment and metabolic data collection. IS performed behavioral assays. IS, AH, TH, EO, and SS performed the immunohistochemical studies. IS, DS, NL, and DB-Z analyzed data. DB-Z and IS wrote the original draft and prepared the figures. All authors contributed to the article and approved the submitted version.
